# Whole exome sequencing in childhood-onset lupus frequently detects single gene etiologies

**DOI:** 10.1186/s12969-019-0349-y

**Published:** 2019-07-30

**Authors:** Irit Tirosh, Shiri Spielman, Ortal Barel, Reut Ram, Tali Stauber, Gideon Paret, Marina Rubinsthein, Itai M. Pessach, Maya Gerstein, Yair Anikster, Rachel Shukrun, Adi Dagan, Katerina Adler, Ben Pode-Shakked, Alexander Volkov, Marina Perelman, Shoshana Greenberger, Raz Somech, Einat Lahav, Amar J. Majmundar, Shai Padeh, Friedhelm Hildebrandt, Asaf Vivante

**Affiliations:** 1 Department of Pediatrics B, Edmond and Lily Safra Children’s Hospital, Sackler Faculty of Medicine, Sheba Medical Center, Tel-Hashomer, 5265601 Ramat Gan, Israel; 2 Rheumatology Unit, Edmond and Lily Safra Children’s Hospital, Sheba Medical Center, Tel-Hashomer, Israel; 3 Department of Pediatrics A Edmond and Lily Safra Children’s Hospital, Sheba Medical Center, Tel-Hashomer, Israel; 4 Intensive care unit, Edmond and Lily Safra Children’s Hospital, Sheba Medical Center, Tel-Hashomer, Israel; 5 Metabolic Disease Unit, Edmond and Lily Safra Children’s Hospital, Sheba Medical Center, Tel-Hashomer, Israel; 6 Pathology Department, Sheba Medical Center, Tel-Hashomer, Israel; 7 Department of Dermatology, Sheba Medical Center, Tel-Hashomer, Israel; 8 Sackler Faculty of Medicine, Tel-Aviv University, Tel-Aviv, Israel; 9 Division of Nephrology, Department of Medicine, Boston Children’s Hospital, Harvard Medical School, Boston, MA USA; 10 The Genomic Unit, Sheba Cancer Research Center, Sheba Medical Center, Tel Hashomer, Israel; 11 Nephrology Unit, Edmond and Lily Safra Children’s Hospital, Sackler Faculty of Medicine, Sheba Medical Center, Tel Hashomer, 5265601 Ramat Gan, Israel

**Keywords:** WES, SLE, Monogenic

## Abstract

**Background:**

Systemic lupus erythematosus (SLE) comprise a diverse range of clinical manifestations. To date, more than 30 single gene causes of lupus/lupus like syndromes in humans have been identified. In the clinical setting, identifying the underlying molecular diagnosis is challenging due to phenotypic and genetic heterogeneity.

**Methods:**

We employed whole exome sequencing (WES) in patients presenting with childhood-onset lupus with severe and/or atypical presentations to identify cases that are explained by a single-gene (monogenic) cause.

**Results:**

From January 2015 to June 2018 15 new cases of childhood-onset SLE were diagnosed in Edmond and Lily Safra Children’s Hospital. By WES we identified causative mutations in four subjects in five different genes: *C1QC*, *SLC7A7*, *MAN2B1*, *PTEN* and *STAT1*. No molecular diagnoses were established on clinical grounds prior to genetic testing.

**Conclusions:**

We identified a significant fraction of monogenic SLE etiologies using WES and confirm the genetic locus heterogeneity in childhood-onset lupus. These results highlight the importance of establishing a genetic diagnosis for children with severe or atypical lupus by providing accurate and early etiology-based diagnoses and improving subsequent clinical management.

## Background

Systemic lupus erythematosus (SLE) can present with a diverse range of clinical manifestations that result from loss of self-tolerance and immune-mediated organ dysfunction. The American College of Rheumatology (ACR) classification criteria requires four out of 11 criteria for the classification of SLE. The diverse clinical manifestations of lupus presents a challenge for clinicians [1]. Childhood-onset SLE accounts for ~ 15% of cases and may differ phenotypically from adult-onset SLE, as it can be more clinically aggressive [2]. The notion that single gene mutations can cause childhood-onset SLE (“monogenic SLE”) is supported by three findings: (i) SLE can appear with familial aggregation; (ii) monogenic mouse models exhibit SLE like phenotypes [3, 4] and (iii) monogenic childhood syndromes with SLE-like features have been described. Until recently, only a handful of monogenic SLE genes had been described in primarily familial cases with congenital early complement protein deficiencies. With advances in next-generation sequencing, ~ 35 single gene mutations causing SLE/SLE-like syndromes have been discovered in humans with recessive and/or dominant modes of inheritance [5, 6]. Given this broad genetic and phenotypic heterogeneity and the rapidly evolving sequencing technology, it is likely that many novel genes will be identified in the near future [5, 6].

Here we report four unrelated severe cases of childhood-onset SLE secondary to mutations in five different genes: *C1QC*, *SLC7A7*, *MAN2B1*, *PTEN* and *STAT1*. We discuss novel clinical insights gained from the genetic discovery in each case, summarize current knowledge of monogenic forms of SLE and suggest clinical features which should alert clinicians to suspect monogenic etiology in SLE patients.

## Methods

### Study participants

After informed consent we obtained clinical data, blood samples, and pedigrees from individuals participating in this study. Approval for research on humans was obtained from Sheba Medical Center and the Boston Children’s Hospital Review Boards. The diagnosis of SLE or SLE like disease was made by a pediatric rheumatologist and met the ACR classification criteria for SLE [1].

### Whole-exome sequencing

Whole exome sequencing (WES) was performed using genomic DNA isolated from blood lymphocytes and later processed using Agilent SureSelect human exome capture arrays (Life Technologies™) with next generation sequencing on an Illumina™ sequencing platform at the Broad Institute (Cambridge MA) and Yale Center for Mendelian Genomics (New Haven, CT). Sequence reads were mapped to the human reference genome assembly (NCBI build 37/hg19 www.genome.ucsc.edu) using CLC Genomics Workbench (version 6.5.1) software (CLC bio, Aarhus, Denmark) as previously described [7]. WES was also performed at the Sheba Hospital Genomic Unit, using an Agilent v5 Sureselect capture kit and Illumina 2500 sequencing technology. For each sample, paired end reads (2 × 100 bp) were obtained, processed and mapped to the genome. We used the BWA mem algorithm (version 0.7.12) [8] for alignment of the sequence reads to the human reference genome (hg19). The HaplotypeCaller algorithm of GATK version 3.4 was applied for variant calling, as recommended in the best practice pipeline. KGG-seq v.08 was used for annotation of identified variants, and in-house scripts were applied for filtering based on family pedigree and local dataset of variants detected in previous sequencing projects.

### Variant calling

Following WES, genetic variants were first filtered to retain only non-synonymous and splice variants. Second, filtering was performed to retain only alleles with a minor allele frequency (MAF) of < 0.01. MAF was estimated using combined datasets incorporating all available data from the 1,000 Genomes Project, the Exome Variant Server (EVS) project, dbSNP142, and the Exome Aggregation Consortium (ExAC). Third, observed sequence variants were analyzed using the UCSC Human Genome Bioinformatics Browser for the presence of paralogous genes, pseudogenes, or misalignments. Fourth, we scrutinized all variants within the sequence alignments of the CLC Genomic Workbench™ software program for poor sequence quality and for the presence of mismatches that indicate potential false alignments. Fifth, we employed web-based programs to assess variants for evolutionary conservation, to predict the impact of disease candidate variants on the encoded protein, and to determine whether these variants represented known disease-causing mutations. Mutation calling was performed by a team of clinician scientists, who had knowledge of the clinical phenotypes and pedigree structure, as well as genetic expertise in homozygosity mapping and exome evaluation as previously described [7]. Sanger sequencing was performed to confirm the remaining variants in original DNA samples and when available to test for familial segregation of phenotype with genotype.

## Results

From January 2015 to June 2018 overall 15 new cases of childhood-onset SLE were diagnosed in our institute. Six out of the 15 newly diagnosed patients, underwent genetic testing given a severe (life-threatening or organ-threatening presentation), atypical presentation (clinical features out of the typical clinical classification criteria for SLE), consanguineous parents or additional comorbidities. We identified causative mutations in four out of these six patients (66%).

### Family 1

**Fig. 1:**
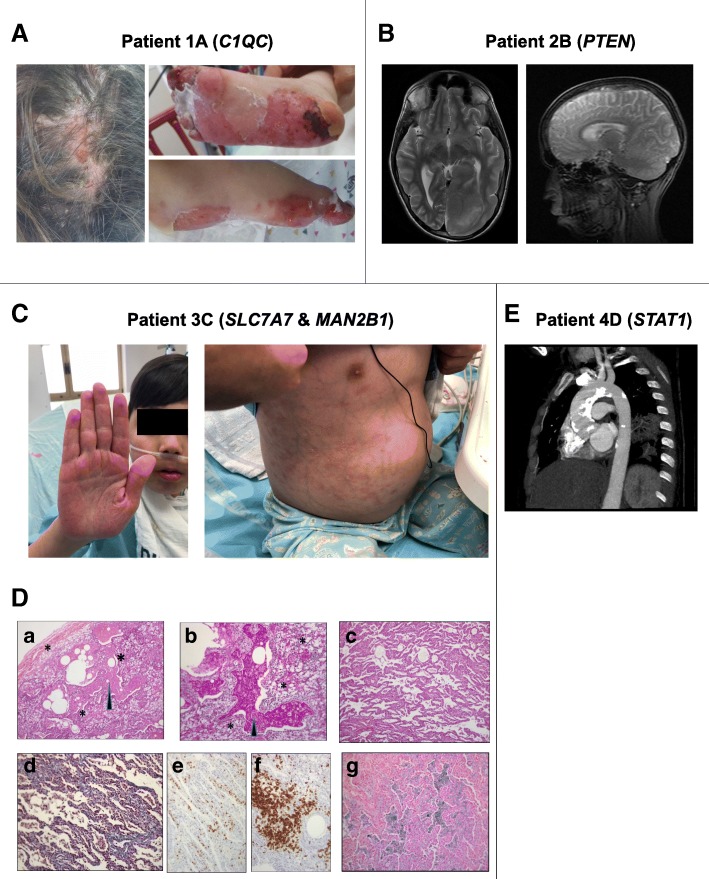
Representative clinical findings in patients with monogenic SLE. Panel **A** – Occipital and lower extremities lesions noted in patients 1A with *C1QC* mutation, reveals palmoplantar erosive erythemic plaques and scarring alopecia. Panel **B** – Abnormal brain MRI scan of patient 2B with *PTEN* mutation shows non-specific parieto-occipital lesions affecting the white matter and cortical dysplasia. Panel **C** – Patient 3C with *SLC7A7* and *MAN2B1* mutations, exhibits a palmar erythema and diffuse abdominal papulosquamous rash. Panel **D** – Lung wedge biopsy of patient 3C shows: (a) sheets of large vacuolated macrophages in the interstitium (asterisks) and eosinophilic intra-alveolar exudate (arrow) H&E X 100. (b) The intra-alveolar exudate contains cholesterol clefts and it is PAS-positive and diastase resistant (PAS-diastase stain X 200). (c) Mild- to moderate alveolar wall thickening (H&E × 100). (d) Mild fibrosis (Masson trichrome stain × 100). (e) Diffuse inflammatory T-cell infiltration (CD3 immunostain × 200). (f) B- cell aggregates (CD20 immunostain × 200). (g) Part of alveolar spaces contain hemosiderin laden macrophages (Prussian blue stain × 200). Panel **E** – Chest CT angiography in patient 4D with *STAT1* gain of function mutation, shows dilated ascending aorta with severe calcifications

Index patient 1A was the youngest daughter of first degree cousins of Muslim ancestry. She presented to an outside hospital at the age of 18 months with a photosensitive rash, oral ulcers, arthralgia, hypertension and Raynaud’s phenomenon. Laboratory testing showed positive ANA and anti-dsDNA serologies. She fulfilled five criteria out of 11 of the ACR classification criteria (oral ulcers, photosensitivity, hematologic disorder, immunologic disorder, and positive antinuclear antibody) and was diagnosed clinically with SLE (Table 1**,** Fig. 1). She was treated with oral corticosteroids with some improvement. At the age of 2 years, during tapering of steroid therapy, she developed daily fever, malar rash, severe digital and oral ulcers, arthritis and gastrointestinal bleeding. As a result she was transferred to our hospital. Upon admission, she had cardiac arrest requiring intubation and prolonged resuscitation. Physical examination revealed classic malar rash, palmoplantar erosive erythematous plaques and scarring alopecia (Fig. 1). Laboratory investigation showed pancytopenia, decreased renal function test and elevated transaminases (Table 1), hyperferritinemia 12,139 ng/mL, hypofibrinogenemia 119 mg/dL, hypertriglyceridemia 349 mg/dL and elevated transaminases. Urinalysis was significant for hematuria and nephrotic range proteinuria. Additional work-up demonstrated a large pericardial effusion, brain hemorrhages and upper gastrointestinal bleeding.

**Table 1:** Laboratory and serology characteristics of affected probands with monogenic childhood-onset lupus

Pt	Hb g/dl	WBC k/uL	ALC k/uL	PLT k/uL	Cr mg/dl (normal range)	Urine Protein/Cr Ratio (< 0.2 g/gr)	AST IU/I 0–60	ALT IU/I 7–45	ESR mm/hr	CRP mg/l 0–5	C3 mg/dl 90–180	C4 mg/d 10–40	IgG mg/d 720–1560	ANA	Anti-dsDNA IU/ml 0–4.99	Anti-smith U/ml 0–20
A1	5.31	1.17	0.73	42	1.03 (0.15–0.37)	6.9	1710	228	22	1.07	61.8	11.8	NA	POS	NEG	21.4
B2	11	3.05	0.59	217	0.64 (0.24–0.73)	2.05	87	60	35	140	49.2	5.95	1400	1:80	35	51.7
C3	9.83	6.06	1.24	141	0.72 (0.24–0.73)	0.49	186	64	NA	25.2	46.5	15.4	2910	1:640	11	< 0.3
D4	11.2	3.9	1.08	187	0.39 (0.45–0.75)	NA	101	66	95	17.2	135	18.4	2580	1:2560	5	1.1

*Abbreviations: Hb* hemoglobin, *WBC* white blood cell count, *ALC* absolute lymphocyte count, *PLT* platelets, *Cr* creatinine, *AST* aspartate aminotransferase, *ALT* alanine aminotransferase, *ESR* erythrocyte sedimentation rate, *CRP* c-reactive protein, *ANA* anti-nuclear antibody, *NA* not available

At that point in time, the differential diagnosis included lupus exacerbation, macrophage activation syndrome (MAS) and sepsis. Extensive infectious studies were negative and she was diagnosed with MAS based on prolonged fever, hyperferritinemia, hypofibrinogenemia, hypertriglyceridemia and elevated transaminases. IV pulse steroids and intravenous immunoglobulins (IVIG) were administered but had poor response. She developed multi- organ failure and disseminated intravascular coagulation which precipitated gastrointestinal and retroperitoneal bleeding. She was treated with vasopressors, high-pressure ventilation, peritoneal dialysis, continuous fresh frozen plasma infusions, broad spectrum antibiotics and cyclophosphamide. However, she clinically worsened and, 2 weeks following her admission to our hospital, she passed away from presumed SLE complicated by MAS.

#### Genetic analysis identifies a novel C1QC truncating mutation

Given the early age of onset and family history of consanguinity, we performed WES analysis under the hypothesis that she had a monogenic form of SLE. We identified a novel homozygous truncating mutation in the gene *C1QC* (c.271G > T p.Gly91*) which results in a premature stop codon and is predicted to be deleterious.

### Family 2

Index patient 2B presented at the age of 13 years with an urticarial rash, weight loss and irritability. Past medical history was notable for macrocephaly, seizure disorder, attention deficit hyperactivity disorder and mild developmental delay. He also had history of unexplained generalized lymphadenopathy first noted at the age of 12 years. On physical examination, height and head circumference were above the 90th percentile for age, he was pale and cachectic. He had an urticarial rash on his trunk and limbs, hypertrophic pigmented gums, polyarthritis, hepatosplenomegaly and generalized lymphadenopathy. Rheumatologic laboratories showed positive ANA and anti dsDNA, anti-SM, anti RNP and anti-ribosomal P serology as well as low complement level (Table 1). Urinalysis was normal and antiphospholipid antibodies were negative. Broad Infectious and hematologic laboratory studies were unrevealing. The patient was diagnosed with lupus as he fulfilled four of the 11 ACR classification criteria: arthritis, hematologic abnormalities, immunologic abnormalities, and positive antinuclear serology. His mood changes, headaches, and high anti-ribosomal P titers suggested central nervous system involvement. Several days into his admission he complained of headache and quickly deteriorated into a comatose state, requiring mechanical ventilation. MRI studies showed high signal intensity along the spinal cord and abnormal intensity around the thalamus (Fig. 1). Lumbar puncture demonstrated pleocytosis, high protein level and low glucose levels. Cerebrospinal fluid studies were negative for viral, bacterial, or fungal infections. He was treated with pulse doses of methylprednisolone, intravenous cyclophosphamide and Rituximab, but had a poor response. Mycophenolate mofetil and daily prednisone were prescribed at the maintenance therapy. Three months later, he developed small intestine necrosis which required total resection of his small intestine and prolonged PICU admission. Unfortunately, at the age of 14 years, the patient passed away secondary to *Pseudomonas* sepsis.

#### Genetic analysis identifies a PTEN mutation

Patient 2B had severe clinical presentation with macrocephaly, developmental delay, pigmented gums and pigmented macules of the glans penis. This led to suspected *PTEN* mutation (Phosphate and Tensin homolog gene) hamartoma tumor syndrome (PHTS). Genetic analysis revealed a previously reported [9] truncating *PTEN* mutation (c.697C > T, p.R233X).

### Family 3

Index patient 3C is an eight-year-old boy from a consanguineous family who was admitted to the PICU of our center with acute respiratory insufficiency and impending respiratory failure. Upon admission he was found to have dysmorphic features, decreased breath sounds bilaterally, hepatosplenomegaly, malar rash, diffuse abdominal papulosquamous rash and palmar erythema (Fig. 1). His diagnostic workup (Table 1) was positive for thrombocytopenia and Coombs positive hemolytic anemia. His serologies showed positive ANA, anti-dsDNA, anti-Ro, ANCA-MPO and ASMA titers. He also had laboratory evidence of hypergammaglobulinemia and hypocomplementemia. His infectious studies were only positive for HHV-6 PCR on broncho-alveolar lavage, which did not explain his severe respiratory symptoms nor his systemic organ involvement. Chest CT imaging demonstrated acute pneumonitis and signs of chronic lung disease. The diagnosis of lupus was made on the basis of the following ACR classification criteria: malar rash, hematologic abnormalities, immunologic abnormalities, and positive antinuclear antibody titers. Lupus nephritis was suspected due to elevated serum urea and creatinine levels, proteinuria, hematuria and hypertension. However, renal biopsy was negative for typical kidney histology of lupus and demonstrated only non-specific tubular damage. The patient was treated with corticosteroids, Plaquenil and anti-hypertensive medications with a very good response.

#### Genetic analysis identifies novel *MAN2B1 and SLC7A7* mutations

Because his parents are first-degree relatives and his lupus presentation was atypical we suspected a recessive monogenic disorder and performed WES. We performed a proband-only exome and detected two homozygous mutations in two different genes: *MAN2B1* (Mannosidase Alpha Class 2B Member 1) and *SLC7A7* (Solute Carrier Family 7 Member 7), in which mutations cause alpha-mannosidosis and lysinuric protein intolerance respectively. Interestingly, both monogenic disorders have been previously described with lupus-like presentation [5]. The *MAN2B1* mutation is predicted to cause a substitution of a highly conserved Valine residue at position 65 to methionine (c.C192A, p.V56 M, CADD: 26.8). The SLC7A7 mutation is, similarly, predicted to cause a substitution of a highly conserved serine residue at position 315 to proline (c.T943C, p.S315P, CADD: 23.8). Both substitutions were predicted to be damaging by multiple in silico tools (Sift, Polyphen2, LRT, Provean, MutationAssessor, Fathmm). The variants were absent from gnomAD database, dbSNP, 1000G, ESP6500 and an in-house database.

### Family 4

Index patient 4D, was initially referred to our primary immunodeficiency clinic. She was born to a non-consanguineous parents and, since early infancy, had recurrent infections including recurrent complicated pneumonias, lung abscesses, bronchiectasis and chronic mucocutaneous fungal infections. She also had recurrent sinusitis, purulent otitis, staphylococcal skin abscesses and corneal infections leading to corneal scarring. Immunodeficiency was suspected and a prophylactic treatment with IVIG infusions, antibiotics and anti-fungal treatment were initiated. Furthermore, the patient had recurrent oral and genital ulcers, recurrent episodes of fever and myalgia, generalized lymphadenopathy and hepatosplenomegaly since early childhood. On physical examination, she displayed short stature (below the 3th percentile), mucocutaneous fungal infections with onychomycosis, corneal scarring, severe caries, oral ulcers, genital ulcers, systolic heart murmur 2/6, shortness of breathing, clubbing, voice hoarseness, productive cough, sinusitis, bilateral purulent otitis and hepatosplenomegaly. Laboratories showed Coombs- positive hemolytic anemia, leukopenia, elevated ANA and anti-dsDNA antibody titers, positive antiphospholipid antibodies, elevated IgG and IgM and absence of IgA and IgE (Table 1**).** Consequently, the clinical diagnosis of lupus was made as she fulfilled four out of 11 ACR criteria: oral ulcers, hematologic abnormalities, immunologic findings and positive ANA. Oral prednisone (5 mg) and Plaquenil were initiated with subsequent clinical improvement. Echocardiography revealed severe dilatation of the right coronary artery, mild aortic insufficiency and dilatation of the ascending aorta. CT-angiography confirmed the dilation of the left main coronary but also revealed dilatation of ascending and abdominal aorta with severe calcinosis of vessel walls (Fig. 1) and bilateral bronchiectasis. Immunologic studies revealed severe lymphopenia of both T and B lineages, elevated proportion of double negative T cells (4.6%), and abnormal T cell receptor repertoire. In light of the diagnostic studies, her therapies were augmented with aspirin, mycophenolate-mofetil, and anti-pneumocystis jirovecii prophylaxis.

#### Genetic analysis identifies *STAT1* gain of function mutation

The combination of chronic mucocutaneous candidiasis with autoimmunity raised suspicion of a STAT, related immunodeficiency. Indeed, STAT 1 and STAT3 phosphorylation assays were abnormal. Subsequent WES identified a previously reported [10] *STAT1* gain of function missense mutation (c.862A > G; p.T288A). This mutation affects a highly conserved amino acid, and the substitution is predicted to be damaging by in silico tools (Sift, Polyphen2, MutationAssessor, MutationTaster). The variants were absent in the gnomAD, dbSNP, 1000 genomes, ESP6500 and in-house databases.

## Discussion

In this case series we present four different childhood onset lupus patients with five distinct monogenic mutations. None of the above monogenic syndromes were recognized in our patients on clinical grounds before the genetic work up. Notably, all patients had severe forms of SLE, including 2 mortalities, which prompted genetic analysis. This case series highlights several important clinical insights.

### Monogenic SLE should be suspected in patients with childhood-onset lupus

Since January 2015 we had 15 patients diagnosed with childhood onset lupus (age range 2–18 years) in our institution. Four patients were eventually diagnosed with monogenic lupus in the subset of six patients we performed genetic testing in. This underscores the need for a high index of suspicion for a genetic SLE, especially in patients with severe childhood-onset presentation and familial consanguinity (Table 2). Our results support the notion that atypical or severe clinical presentations may suggest a genetic etiology for SLE. For instance, patient 3C (*MAN2B1* and *SLC7A7)* presented with predominant lung involvement which is an extremely rare manifestation as the first presentation of lupus. Another example is patient 2B (*PTEN*) who presented with longstanding lymphadenopathy, which is also an atypical presentation of lupus. Moreover, patients with childhood lupus with clinical features beyond the clinical spectrum of lupus, such as cases 2B and 3C, should alert clinicians to suspect an underlying genetic SLE etiology. Patient 2B had macrocephaly, developmental delay, high birth weight, pigmented macules on the penis and pigmented gums while patient 3C had significantly enlarged kidneys with renal biopsy findings showing tubular damage. Similarly, in case 4D the concomitant severe immune deficiency was another clinical clue. Lastly, two out of four patients presented here did not respond to the conventional SLE treatment, which in our opinion, should also imply consideration of genetic analysis (Table 2).

**Table 2:** Clinical features that should prompt suspicion for monogenic lupus/lupus-like

Early onset – < 10 years of age	
Suspected (e.g. recurrent infections) or proved immunodeficiency	
Clinical features out of the typical clinical classification criteria for SLE	
Severe, life-threatening or organ-threatening presentation	
Aggressive course, rapid deterioration and/or accumulation of organ damage	
Poor response to treatment	
Familial cases	
Consanguinity	

### Establishing genetic etiology may influence monitoring and treatment

Revealing the molecular genetic diagnosis in patients with childhood-onset lupus can facilitate a personalized medical approach with targeted monitoring and treatment. The first identified, and most described forms of monogenic lupus are inherited complement deficiencies [11] as we identified in Case 1A. It is estimated that the prevalence of autoimmunity with lupus-like manifestations in C1q deficiency is as high as 90%. These conditions predispose to lupus due to impaired tolerance and aberrant clearance of apoptotic bodies and immune complexes [12]. C1q is central in clearing apoptotic debris, but when impaired, autoantigens accumulate and stimulate nucleic acid autoantibodies. Confirming this diagnosis opens a window of opportunity for specific treatments such as fresh frozen plasma or hematopoietic stem cell transplantation [13], which are not part of the conventional lupus treatment and should be considered early in management.

In case 3C we detected two different metabolic diseases: Lysinuric protein intolerance (LPI) caused by mutations in *SLC7A7* and Alpha-mannosidosis caused by mutations in *MAN2B1*. LPI is an autosomal recessive transport disorder of the dibasic amino acids lysine, arginine and ornithine in the renal tubules, intestinal epithelium, hepatocytes and fibroblasts [14]. Deficiency of arginine and ornithine impairs the function of the urea cycle, causing hyperammonemia. There are few case reports of LPI patients who developed SLE and the pathophysiology is not well understood. However, Lukkarinan et al. showed that the humoral immune responses in some patients with LPI may be defective [14]. Alpha-mannosidosis is caused by deficiency of lysosomal alpha-mannosidase (LAMAN). Three major clinical subtypes have been suggested [15] with various severities of skeletal abnormalities and myopathy and neurological manifestations. Associated medical problems may also include corneal opacities, hepatosplenomegaly, aseptic destructive arthritis. The association between alpha mannosidosis and lupus has been reported in the past in several case reports [16].

Each of the above mentioned syndromes can present with SLE like symptoms. This made the clinical diagnosis in patient 3C challenging. Hence, this unique situation of patients from consanguineous families harboring two different disease causing mutations should always be considered by clinicians [17]. Specific treatments for these genetic diseases include enzyme replacement therapy for mannosidosis [18] and low protein diet with supplementation of citrulline for LPI. Identifying the genetic diagnosis may better define which of the patient’s clinical symptoms can be attributed to autoimmunity as opposed to symptoms arising secondary to the metabolic abnormality, and therefore guide the treatment. Thus, ascribing the severe lung disease in case 2 to lupus-related lung involvement (e.g. pneumonitis) may require maximal immunosuppressive therapy. However, diagnosing the lung disease as part of the LPI presentation which was supported by the patient’s lung histology findings (Fig. 1) mandates a completely different treatment approach and may prevent unnecessary procedures and treatments.

Genetic diagnosis may additionally guide disease specific monitoring. Patients diagnosed with autosomal dominant *PTEN* mutations (a known tumor suppression gene) have high risk for benign and malignant tumors of the thyroid, breast, and endometrium, as well as for neurodevelopmental disorders. Additionally, PTEN was found to be important for proper T regulatory cell functioning and autoimmunity prevention [19]. These observations, as well as the *Pten* mice models [20] support that a lupus-like phenotype can be caused by *PTEN* mutations.

Similarly, patients with complement deficiencies or *STAT1* mutations should be monitored for severe bacterial infections [21]. Heterozygous gain of function mutations in *STAT1* lead to impaired nuclear dephosphorylation of STAT1 and immune aberrations which include lymphopenia, reduced responses to mitogens and antigens, hypogammaglobulinemia, as well as impaired natural killer (NK) cell function. Clinical manifestations in patients with *STAT1* mutation, in addition to immunodeficiency includes inflammatory and autoimmune phenomena such as hypothyroidism (22%), type 1 diabetes (4%), blood cytopenia (4%), and SLE (2%) [21, 22]. Rarely, patients can have cerebral vasculitis and multiple aneurysms leading to stroke [23]. Aortic calcifications and aneurism were also reported [24]. Specific treatments including prophylactic antifungal and antimicrobial agents, IVIG, and recently the utility of JAK inhibitors in these patients has been suggested [25].

### Genes mutated in monogenic forms of lupus converge to signaling pathways that inform disease pathogenesis

Over the last decade the growing use of whole exome sequencing revealed additional culprit genes leading to human monogenic forms of lupus resulting in better understanding of pathogenic pathways. These pathways can be grouped as follows [5, 12]: [1] Complement; [2] Apoptosis and nucleic acid degradation, repair and sensing; [3] Type I interferon pathway; [4] B cell and T cell tolerance, and [5] other (Table 3). Moreover, accounting for additional genes described in monogenic forms of lupus in mouse models, it is likely that many more remain to be identified (Table 4).

**Table 3:** Single gene causes of lupus or lupus-like syndrome in Humans

Mechanism	Gene Symbol	Protein	MOI	Phenotype	Ref	Human Disease [OMIM#]
Complement	*C1QA*	C1Q	AR	SLE in 88% Recurrent infections	[26]	120550
	*C1QB*		AR		[27]	120570
	*C1QC*		AR		[28]	120575
	*C1R*	C1R	AR	SLE in 65% Sjogren syndrome Recurrent infections	[29]	613785
	*C1S*	C1S	AR		[30]	120580
	*C2*	C2	AR	SLE in 10% Recurrent infections	[31]	613927
	*C3*	C3	AR	SLE in a minority of affected	[32]	120700
	*C4*	C4	AR	SLE in 75% Recurrent infections	[33]	142974
Type 1 interferon	*TMEM173*	STING	AD	STING associated vasculopathy with onset in infancy	[34]	612374
	*SAMHD1*	SAMHD1	AR	Mild Aicardi–Goutie` res syndrome Mouth ulcers Deforming arthropathy Cerebral vasculopathy	[35]	606754
	*ADAR1*	ADAR1	AR/AD	Aicardi–Goutie’res syndrome Bilateral striatal necrosis	[36]	146920
	*IFIH1*	IFIH1	AD	Classical or mild Aicardi–Goutie’res syndrome Singleton–Merton syndrome SLE	[36]	606951
	*RNASEH2B*	RNASEH2B	AR	Aicardi–Goutie’res syndrome	[36]	610326
	*APC5*	APC5	AR	SLE Sjogren syndrome Autoimmune cytopenias Raynaud phenomenon Recurrent infections Spondyloenchondrodysplasia	[37]	606948
	*TREX1*	TREX1	AR	Aicardi–Goutie’res syndrome	[36]	606609
Nucleic acids degradation	*DNASE1*	DNASE1	AD	SLE Sjogren syndrome	[38]	125505
	*DNASE1L3*	DNASE1L3	AR	SLE Hypocomplementemic urticarial vasculitis syndrome	[39]	602244
	*TREX1*	TREX1	AD	Aicardi–Goutie’res syndrome	[40]	606609
	*RNASEH2A*	RNASEH2A	AR	Aicardi–Goutie’res syndrome	[41]	606034
RAS/MAPK	*SHOC2*	SHOC2	AD	Noonan syndrome with loose anagen hair SLE	[42]	602775
	*KRAS*	KRAS	AD	Noonan syndrome SLE	[42]	190070
	*PTPN11*	PTPN11	AD	Noonan syndrome SLE (polyarthritis, photosensitivity, leukopenia and lymphopenia) Hashimoto thyroiditis	[42]	176876
Proteasome	*PSMA3*	PSMA3	AD	CANDLE (chronic atypical neutrophilic dermatosis with lipodystrophy and elevated temperature)	[43]	176843
	*PSMB4*	PSMB4	AD		[43]	602177
	*PSMB8*	PSMB8	AD		[44]	177046
Apoptosis	*TNFRSF6*	FAS	AD	ALPS	[45]	134637
	*FASLG*	FASL	AD	ALPS SLE with lymphoadenopathies	[8]	134638
Tolerance	*PRKCD*	PRKCD	AR	SLE (Malar rash & nephritis 100%)	[46]	176977
	*RAG2*	RAG2	AR/AD	SCID Omenn syndrome SLE	[47]	179616
Phagocytes oxidase system	*CYBB*	NADPH oxidase 2	X-linked	Chronic granulomatous disease Cutaneous lupus erythematosus SLE	[48]	300481
DNA repair	*NEIL3*	NEIL3	AR	Autoimmune cytopenias Chronic diarrhea Recurrent Infections	[49]	608934
AKT/PKB	*PTEN*	PTEN	AD	SLE Malignancy Bannayan–Riley–Ruvalcaba syndrome Cowden syndrome	[50]	601728
Collagen degradation	*PEPD*	PEPD	AR	Prolidase deficiency Leg ulcers SLE	[51]	613230
Amino acid transporter	*SLC7A7*	SLC7A7		Lysinuric protein intolerance SLE	[52]	603593
Carbohydrate catabolism	*MAN2B1*	Lysosomal α mannosidase	AR	Alpha-mannosidosis SLE	[16]	609458

**Table 4:** Mouse models of lupus

	Gene	Reference
1	C1qa	[53]
2	C4b	[54]
3	Cd40lg	[55]
4	Cdkn1a	[56]
5	Def6	[57]
6	Dnase1	[58]
7	Ep300	[59]
8	Fas	[59]
9	Fcgr2b	[60]
10	Gadd45a	[56]
11	Ifih1	[61]
12	Ikzf3	[62]
13	Jak1	[63]
14	Junb	[64]
15	Lbr	[65]
16	Lyn	[66]
17	Man2a1	[67]
18	Mta2	[68]
19	Pdcd1	[69]
20	Polb	[70]
21	Pparg	[71]
22	Prdm1	[72]
23	Ptprc	[73]
24	Rasgrp1	[74]
25	Rassf5	[75]
26	Rc3h1	[76]
27	Rxra	[71]
28	Trl7	[77]
29	Tnfrst13b	[78]
30	Traf3ip2	[79]
31	Trove2	[80]

## Conclusions

Our findings demonstrate a significant detection rate for monogenic etiologies using WES and reveal broad genetic heterogeneity in clinically complex cases of childhood-onset lupus.

These results highlight the importance of genetic diagnosis especially for children with severe or atypical presentations as well as for familial cases and individuals from consanguineous families. Pursuing WES as part of the diagnostic approach in specific cases of childhood-onset lupus (Table 2), provides opportunities for an accurate and early etiology-based diagnosis which can improve clinical management. Specifically, it may allow gene based multidisciplinary team approach and may lead to identifying additional affected family members who can be asymptomatic or present with subtle clinical findings. An unbiased genetic screening of larger cohorts of patients with childhood-onset SLE with diverse clinical presentations is needed to better estimate the prevalence of monogenic etiology for pediatric SLE.

## Data Availability

The datasets used in the current study are available from the corresponding author on reasonable request.

## Abbreviations

ACR: The American college of rheumatology
EVS: Exome variant server
ExAC: Exome aggregation consortium
IVIG: Intravenous immunoglobulins
LPI: Lysinuric protein intolerance
MAF: Minor allele frequency
MAS: Macrophage activation syndrome
SLE: Systemic lupus erythematosus
WES: Whole exome sequencing
